# Affordable Imaging Lab for Noninvasive Analysis of Biomass and Early Vigour in Cereal Crops

**DOI:** 10.1155/2018/5713158

**Published:** 2018-04-19

**Authors:** Rita Armoniené, Firuz Odilbekov, Vivekanand Vivekanand, Aakash Chawade

**Affiliations:** ^1^Department of Plant Breeding, Swedish University of Agricultural Sciences, 23053 Alnarp, Sweden; ^2^Centre for Energy and Environment, Malaviya National Institute of Technology, Jaipur, Rajasthan 302017, India

## Abstract

Plant phenotyping by imaging allows automated analysis of plants for various morphological and physiological traits. In this work, we developed a low-cost RGB imaging phenotyping lab (LCP lab) for low-throughput imaging and analysis using affordable imaging equipment and freely available software. LCP lab comprising RGB imaging and analysis pipeline is set up and demonstrated with early vigour analysis in wheat. Using this lab, a few hundred pots can be photographed in a day and the pots are tracked with QR codes. The software pipeline for both imaging and analysis is built from freely available software. The LCP lab was evaluated for early vigour analysis of five wheat cultivars. A high coefficient of determination (*R*^2^ 0.94) was obtained between the dry weight and the projected leaf area of 20-day-old wheat plants and *R*^2^ of 0.9 for the relative growth rate between 10 and 20 days of plant growth. Detailed description for setting up such a lab is provided together with custom scripts built for imaging and analysis. The LCP lab is an affordable alternative for analysis of cereal crops when access to a high-throughput phenotyping facility is unavailable or when the experiments require growing plants in highly controlled climate chambers. The protocols described in this work are useful for building affordable imaging system for small-scale research projects and for education.

## 1. Introduction

Phenotyping morphological and physiological traits of plants is one of the most laborious tasks in plant breeding and thus automated high-throughput plant phenotyping (HTP) facilitates measurement of such traits. Visible light (RGB) imaging facilitates measurement of plant's morphological traits such as biomass, height, width, color, number of leaves, and roots to estimate plant growth rate, health, nutrition status, drought stress, water-use efficiency, nutrient-use efficiency, and early vigour [1–4]. 3D imaging can additionally measure traits such as leaf angle and leaf area which affect photosynthesis efficiency of the plants [5–7]. Hyperspectral, thermal, near-infrared (NIR), and fluorescent imaging are useful for detecting abiotic and biotic stresses [8–12]. While RGB imaging is a common feature in most facilities, some also offer fluorescence, thermal, NIR, or UV imaging. 3D imaging is popular and is available at bigger facilities such as Agrobios Plant Scanalyzer (APS) facility in Italy and The Plant Accelerator-Australian Plant Phenomics Facility. Several freely available software programs are available for image analysis [13] such as HTPheno [14], PlantCV [3], Easy Leaf Area [15], Integrated Analysis Platform [16], ImageHarvest [17], and Canopeo [18].

Whole plant biomass and growth rate at the seedling stage are traits that correlate well with early vigour and can be estimated by HTP in cereal crops. Higher early vigour is associated with higher water-use efficiency [19], nitrogen and phosphate uptake [20, 21], and weed competition [22]. 3D imaging with a NIR camera was used to measure early vigour traits such as leaf length and width and tillering in wheat [5]. Thus, HTP can aid in the evaluation of plants for early vigour based on plant biomass and growth rate both in the controlled conditions [17, 20, 23–26] and in the field [4, 26–28].

Major limiting factors for HTP in the controlled conditions are access to an imaging facility or the costs for establishing one. Thus, such facilities are established with the aim of providing phenotyping as a service and when a high-throughput and continuous use of the facility is anticipated. A low-cost phenotyping facility could be a viable alternative when (i) access to a high-throughput facility is unavailable, (ii) phenotyping occasionally for small-scale projects, and (iii) phenotyping is done in climate chambers with constrained spaces. Several custom made affordable imaging systems have been developed for studying drought stress in wheat [29] biotic stress and magnesium deficiency in common beans [30], cold tolerance in pea [31], and biomass in sorghum [32]. A review of various imaging systems and the studied traits was recently published [1]. To be cost-effective, sustainable, and time efficient, a low-cost phenotyping system must produce reproducible results with a reasonable amount of manual labor for setting up, running, and management of the system.

In this work, we have set up a low-cost RGB imaging phenotyping lab that includes automated plant tracking using QR code, imaging, and image analysis. The instructions for setting up a low-cost imaging lab and the data analysis pipeline are described. This system is demonstrated with early vigour analysis in wheat.

## 2. Materials and Methods

### 2.1. Setting Up an Imaging Studio

**Figure 1:**
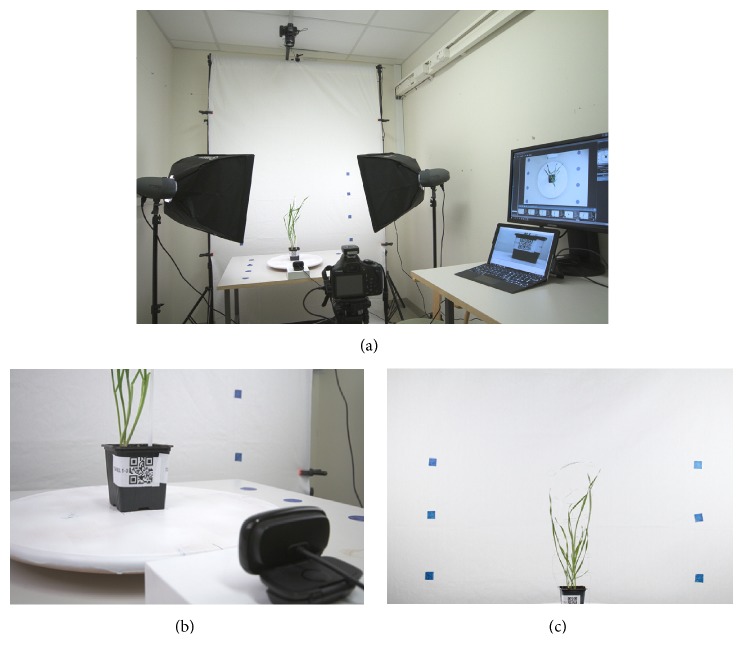
Photography setup for low-cost imaging lab. (a) Sample is placed on a rotating disc and is evenly light by two studio strobes. Images are taken with a side-view and a top-view digital camera. (b) QR code on the pot is read by a webcam. All three cameras are tethered to a computer. (c) An exemplified image.

The low-cost phenotyping lab (LCP lab) consists of two studio strobes (Visico ELFIN VL-200 Plus) with an effect of 200 W each and color temperature of 5600 K. The two strobes are fitted with softboxes (50 × 70 cm) on light stands and placed one on each side of the plant at an angle of 45° illuminating both the plant and the background. The strobes contain integrated wireless radio receivers and the images are triggered with a wireless radio transmitter (V801TX, Visico, China) connected to a camera. The white background (1.5 × 2.5 m; FotoBestway Co., Ltd.) is hung on a telescopic boom placed on two light stands one on each side. Blue markers are pasted on the background to aid in framing of the images. Imaging is performed with two entry-level digital single lens reflex (DSLR) cameras (Canon 1300D, Canon, USA) and the 18–55 mm kit lens. The side-view camera is mounted on a tripod, while the top-view camera is mounted on a SpaceArm (Tristar). The distance between the side and the top-view camera and the pot is 1.5 m. A pot is placed on a rotating disc of diameter 38 cm (Snudda, IKEA) which is spray painted to white. Optionally, the pot can also be placed on a rotating turntable of similar diameter. For reading the QR code from the pot, a webcam (Logitech International S.A., USA) is placed 20 cm away from the pot. All three cameras are connected to a computer and tethered (Figure 1).

**Figure 2:**
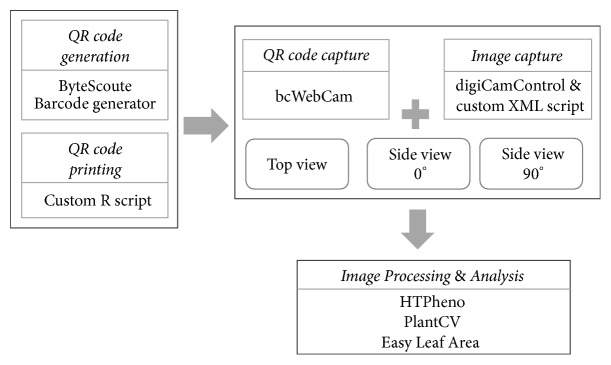
Pipeline for imaging and data processing. QR codes are generated with ByteScoute and printed with a custom R script. Tethered imaging is done with digiCamControl with custom XML script for time-lapse imaging and QR code capture with bcWebCam. Image processing and analysis are done with HTPheno, PlantCV, and Easy Leaf Area software.

Pots can be tagged with QR code containing desired metadata such as the cultivar name, replicate number, and treatment. QR code is generated with Bytescout Barcode generator (https://www.bytescout.com) and printed on self-adhesive labels using a custom R script (Supplementary File 1). Once a pot is placed on a rotating disc, the QR code is read by the webcam placed in front of the pot and operated by the software bcWebCam (http://www.bcwebcam.de). The information read from the QR code is automatically transferred from bcWebCam to the tethering software digiCamControl (http://www.digicamcontrol.com) (Figure 2). The two DSLR cameras are tethered to digiCamControl which takes a series of three images using a custom XML script (Supplementary file 2) such that the first image is taken by the side-view camera followed by a one-second wait and a second image by the top-view camera followed by a six-second wait to manually rotate the disc with pot by 90° and a third image is taken again by the side-view camera. This imaging series is set in a loop in the XML script which can trigger the cameras for a designated number of times. Side-view images are taken with the camera with optimum settings (focal length of 43 mm, ISO 400, F-Stop f/10, and exposure time of 1/100 seconds), while the top-view images are taken with slightly different settings (focal length of 43 mm, ISO 400, F-Stop f/11, and exposure time of 1/60 seconds).

It is important to evenly light the plants and the background to avoid shadows caused by uneven lighting. Strong shadows were avoided in the images and were controlled by adjusting camera settings and exposure. Oversaturation of the images was avoided by referring to the histograms. Upon optimizing lighting, camera settings, and camera distance, custom white balance was obtained by photographing just the background. All images were thereafter captured with the custom white balance to avoid variation in the white balance and light intensity in the images. The images were stored directly to the computer and are of resolution 72 dpi, size 1920 × 1280 pixels in JPEG format, and the files are named with the data read from the QR code. The pots were black in color and did not interfere with image processing.

### 2.2. Image Processing

Images are first manually inspected to remove those that are improperly lit, plants being out of frame or blurred. Finally, only plants with all three images are retained for further analysis. Thereafter, the images are processed with the software HTPheno [14], PlantCV [3], or Easy Leaf Area [15] on a laptop with a dual-core i7 Intel processor and 16 GB RAM. Optimization of image analysis parameters was carried out for each software program. For HTPheno, the object classes for the side and top-view were background, pot, stickers, and the plant. Thereafter, for each object, the corresponding image coordinates were assigned separately for the top and the side-view. The color ranges for the four objects were assigned with HTPcalib. The PlantCV analysis pipeline was built as described previously [3]. Briefly, (i) conversion of RGB images to HSV color space, (ii) isolation and thresholding the saturation channel, (iii) conversion of RGB to LAB color space and isolation and thresholding of the blue-yellow channel, (iii) joining the saturation and the blue-yellow channels to mask the RGB image followed by extraction and thresholding of green-magenta and blue-yellow channels, and (iv) joining saturation and blue-yellow channels, masking the previously masked image and object identification. For Easy Leaf Area, parameters were leaf minimum green RGB value: 23; G/R: 1.0; G/B: 1.01; scale minimum red RGB value: 33; scale red ratio: 1.0; processing speed: 2.0; minimum leaf pixel: 300.

### 2.3. Plant Material for the Case Study

Five winter wheat cultivars Stigg, Kranich, Nelson, Nimbus, and Target were chosen for early vigour evaluation as these are popular varieties for commercial cultivation. The seeds were germinated for two days on a moist filter paper in Petri dishes. Germinated seeds were sown in plastic pots (0.4 l) filled with peat substrate Blomjord Exclusive (Emmaljunga Torvmull AB, Sweden). One seed per pot of each genotype was sown in ten replications for each time point. Plants were grown at 20°C in a greenhouse with 16 h photoperiod and light intensity of 250 *μ*mol m^2^ s^−1^. All pots were soaked in equal quantity of water every three days. Seedling images were taken at 10 and 20 days upon sowing. The plants were photographed from two side views and one top view. For each timepoint, the first images were taken by including a ruler to adjust for any changes in the camera distance. The conversion from pixels to centimeter was performed separately for 10 and 20 days timepoint and for two centimeter distance, it was 51 pixels for the side view and 53 pixels for the top view for the two timepoints, respectively. For dry weight analysis, after imaging, the shoots were cut and wrapped in aluminum foil then dried at 100°C for 48 h and weighed. Images were manually filtered out to remove those with plants out of frame. From the 10-day timepoint, no images were removed but at the 20-day timepoint, one plant sample each from Stigg, Target, and Nimbus and two plant samples from Nelson had to be removed due to plants being out of frame in the top view. Thus, for the 10-day timepoint, there were 10 replicates each, while there were 8 replicates for the 20-day timepoint.

### 2.4. Statistical Analysis

Relative growth rate for each plant based on the dry weight (RGR_DW_) [23, 33] was estimated with (1), where *W*_1_ and *W*_2_ are dry weights of each plant at timepoints *t*_1_ (10 days) and *t*_2_ (20 days), respectively.

(1) $$\begin{array}{c} RGR_{DW}=\frac{\ln\left(W_{2}/W_{1}\right)}{t_{2}-t_{1}}. \end{array}$$

Projected leaf area (PA) is defined by (2), where PA is the projected leaf area obtained from each image and *n* is the number of angles photographed for a given plant

(2) $$\begin{array}{c} PA=\overset{n=3}{\underset{1}{\sum }}pa. \end{array}$$

Relative growth rate for each plant based on the projected leaf area (PA) was estimated with (3) where PA_1_ and PA_2_ are the projected areas of plants at timepoints *t*_1_ (10 days) and *t*_2_ (20 days), respectively.

(3) $$\begin{array}{c} RGR_{PA}=\frac{\ln\left({PA}_{2}/{PA}_{1}\right)}{t_{2}-t_{1}}. \end{array}$$

## 3. Results

The aim of this work was to build a RGB imaging based phenotyping system that is affordable and portable for low-throughput imaging. LCP lab integrates three cameras, two for imaging and one for reading the QR code (Figure 1). The measurements obtained from the three software programs vary and thus the selection of the software depends on the overall goal of the experiment. HTPheno generates plant height, width, and projected shoot area from the side-view images and *x*-extent, *y*-extent, diameter, and projected shoot area from the top-view images. PlantCV generates over 30 different measurements. Easy Leaf Area estimates the projected leaf area in both side- and top-view images. Measurements from the side and top views can be integrated prior to further analysis.

### 3.1. Case Study: Early Vigour Analysis in Wheat

**Figure 3:**
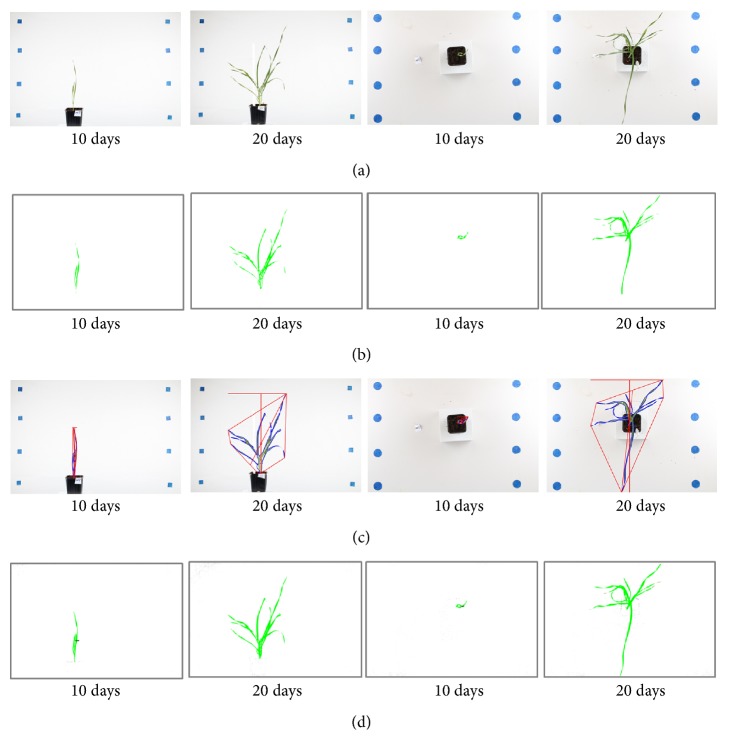
Side- and top-view images of cultivar Nelson at three timepoints after sowing. Images are (a) unprocessed or processed by software (b) HTPheno, (c) plantCV, or (d) Easy Leaf Area.

To estimate the accuracy of imaging with the LCP lab, early vigour analysis was evaluated from 10–20-day-old wheat plants from five cultivars. Images were analyzed with three different software programs HTPheno, plantCV, and Easy Leaf Area. The parameters were adjusted for each software program to maximize the leaf area detection and minimize the detection of other non-plant objects in the images. The three software programs evaluated here have led to an output consisting of a text file with the measurements and images marked with the identified plant regions (Figure 3).

**Figure 4:**
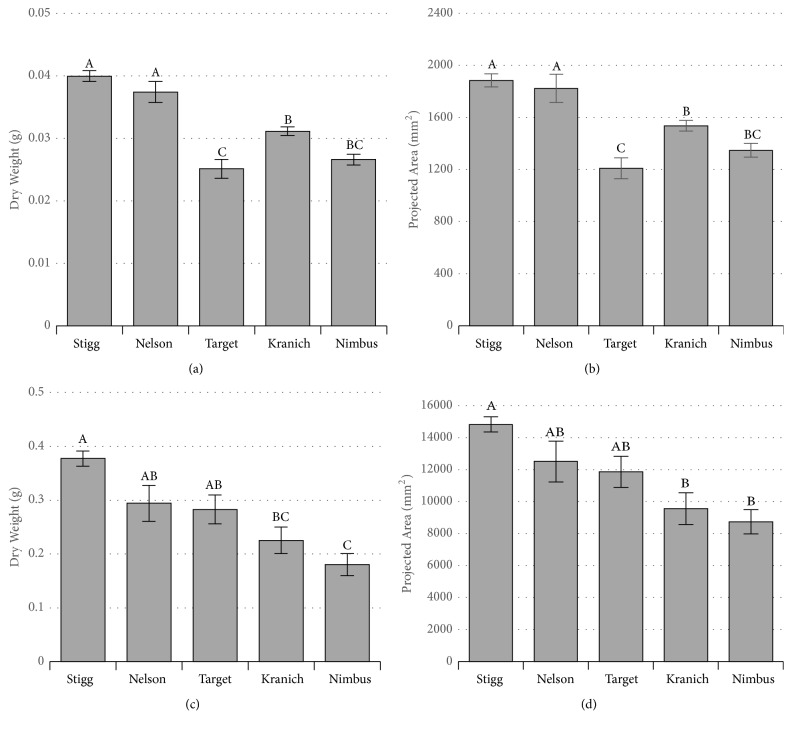
Mean dry weight and mean area of the plants from five cultivars. (a, c) Mean dry weights plants from five cultivars. (b, d) Mean projected leaf area of plants from five cultivars. (a-b) 10 days (*n* = 10); and (c-d) 20 days (*n* = 8) after sowing. Cultivars are sorted based on the mean growth at 20 days. Statistically significant differences (Tukey's HSD adjusted *p* < 0.05) in the means are denoted by different letters above the bars. Error bars are standard error.

Analysis of variance (ANOVA) from the dry weight data showed that the effect of the genotype (cultivars) on the early vigour was significant (*p* < 0.001) and that there was a significant difference in the mean weight between Stigg and Kranich and Stigg and Nimbus at both timepoints (Tukey's HSD adjusted *p* < 0.05) (Figures 4(a) and 4(c)). ANOVA from HTPheno projected leaf area (PA) showed that the effect of the genotype on the early vigour was significant (*p* < 0.001) and based on the projected leaf area (PA) from HTPheno results, Stigg had significantly different early vigour compared to Kranich and Nimbus at all timepoints (Tukey's HSD adjusted *p* < 0.05) (Figures 4(b) and 4(d)).

A simple linear regression was calculated to estimate the coefficient of determination between the measured dry weights and the projected leaf area (PA) for each plant (Table 1). Results from HTPheno had the highest *R*^2^ at 20-day timepoint. Overall, all three software programs had higher *R*^2^ at 20 days compared to the 10-day timepoint. To evaluate if imaging of all three angles is required, *R*^2^ was obtained for dry weight and images obtained from each of the angles separately or combinations of any two angles (Table 2). The results show that across all timepoints, higher *R*^2^ is obtained from images from all three angles.

**Figure 5:**
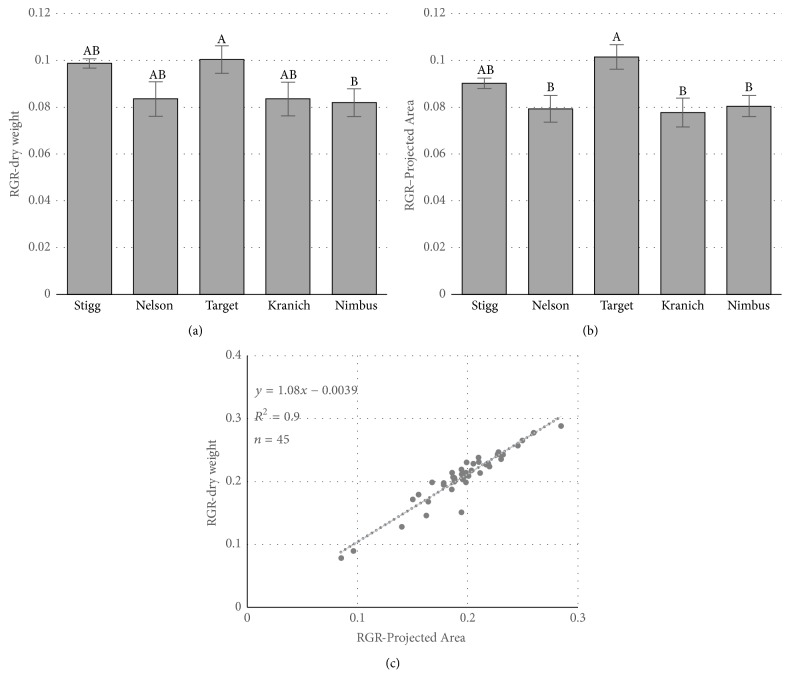
Relative growth rate of the plants from five cultivars based on (a) dry weight and (b) projected leaf area (PA) from HTPheno. Statistically significant differences (Tukey's HSD adjusted *p* < 0.05) in the means are denoted by different letters above the bars. Error bars are standard error. (c) Simple linear regression between RGR_DW_ from dry weight and RGR_PA_ from the projected leaf area from HTPheno.

Relative growth rate estimated from dry weight (RGR_DW_) and HTPheno projected area (RGR_PA_) suggests that the cultivar Target has significantly different growth rate (Tukey's HSD *p* < 0.05) from cultivar Nimbus (Figures 5(a) and 5(b)). A significant regression equation was obtained (*p* < 0.001) between the dry weight and HTPheno projected leaf area. Based on overall growth at 20 days and the relative growth rate analysis, it can be suggested that Stigg has the most growth and higher relative growth rate compared to Nimbus. 

## 4. Discussion

Measurement accuracy and reproducibility are the key factors for the evaluation of a phenotyping pipeline. The highest coefficient of determination (*R*^2^ = 0.94) obtained in this work was with images from 20-day-old plants analyzed with the HTPheno pipeline (Table 1) and for the relative growth rate, *R*^2^ of 0.9 was obtained for dry weight and HTPheno projected leaf area. However, results from plantCV were only slightly lower. PlantCV offers several customizations, takes relatively less time for analysis, and can be a suitable alternative to HTPheno for larger data sets. In a previous study, phenotyping pipeline consisting of a commercial imaging system Scanalyzer 3D (LemnaTec GmbH, Aachen, Germany) and an open source analysis pipeline IAP was used to photograph maize plants and obtained *R*^2^ of 0.84 and 0.94 with the dry and fresh weight, respectively, with RGB imaging [16]. In another study, 373 rice genotypes were photographed with Scanalyzer 3D system and analyzed with the open source ImageHarvest analysis pipeline and obtained *R*^2^ of 0.93 with the shoot dry weight, while with the commercial data analysis pipeline LemnaGrid, *R*^2^ of 0.94 was obtained [17]. Imaging of 320 wheat plants with a Scanalyzer 3D system and analysis with LemnaTec 3D Image Analyzer was done and obtained *R*^2^ of 0.96 with a linear model [34]. 3D imaging of rapeseed with PlantEye F300 developed by Phenospex (Heerlen, the Netherlands) resulted in *R*^2^ of 0.97 with shoot dry weight and 3D leaf area [35]. In the current study, lower *R*^2^ at the earlier timepoint can be attributed to technical errors or lower plant to background ratio due to the smaller size of the plants. Focal distance can be adjusted to increase the plant to background ratio but a correction for the field of view needs to be done prior to comparing data from different timepoints [3].

Out of the three software programs tested here, EasyLeafArea is the easiest to set up and has a graphical user interface and a few parameters to be adjusted. It also detects a red object with known dimensions in an image to estimate absolute measurements of the leaf area automatically. PlantCV requires additional dependencies but the installation is well documented. It does not have a graphical user interface and some knowledge of programming is essential to optimize the program for images taken with a new setup. PlantCV also allows detection of objects with known dimensions and a set of computer code can be written to estimate the absolute measurements by including objects with known dimensions. HTPheno is a plugin for the software ImageJ [36]. Optimization of HTPheno for a new set of images is slightly tedious as the color profiles for the plants need to be manually identified. However, based on this work, the results produced from HTPheno were the best among the three (Table 1). HTPheno also allows estimation of absolute measurements; however, unlike the previous two software programs, in HTPheno, the pixel to centimeter conversion needs to be manually entered. All three software programs are very well documented. Although, in this work, HTPheno performed the best, further optimization of all three software parameters is possible which may improve the results in future studies.

Major advantage of the LCP lab is that it requires just around 10–12 m^2^ of working space. The software digiCamControl supports a range of cameras and although we have used an entry-level DSLR camera Canon 1300D (~350 USD), the total cost can be further reduced with an in-expensive consumer camera model such as Nikon Coolpix S5300 (~200 USD) supported by the digiCamControl. The studio equipment used in this work is easily available worldwide or can be replaced with the equivalent equipment from other brands. More cameras can be added such as those modified for taking Normalized Difference Vegetative Index (NDVI) pictures. In this work, we used a manual rotating disc, but a semi-automated or a fully automated and programmable rotating base can also be installed for further automation of imaging. Pots were tracked with QR codes and read with a webcam and the metadata is stored in the image filename. This enables automated file naming and classification that simplifies the whole image processing. For the presented case study, QR codes were not used but are described here for ease of implementation in building new imaging systems of this kind.

There are several commercial or custom made HTP facilities available featuring RGB, thermal, infrared, or fluorescence imaging as reviewed earlier [1, 11]. These state-of-the-art HTP facilities are capable of imaging hundreds of plants day and night. In some facilities, plant watering and imaging are fully automated through pot weighing and conveyor belts, thus requiring minimal manual labour. These facilities are however expensive to install and maintain requiring considerable investment and resources. Also, these HTP facilities, although they can be modular, still require much bigger working space area mainly for the conveyor belt and are not portable. The proposed LCP lab here although lacks many of the key features available in the large-scale systems, the results obtained in this work suggest that a low-cost system can be a viable option in cases where large-scale facilities are not accessible. The smaller size and portability allow the LCP lab to be installed in smaller walk-in growth chambers which enables imaging plants grown in highly controlled growth conditions. It could also be useful for low-throughput phenotyping projects and/or education.

## 5. Conclusions

We have developed a low-cost RGB imaging phenotyping lab which integrates both imaging and analysis and the detailed description for setting up such a system is provided. LCP lab is a reliable and sustainable option for performing imaging based analysis of morphological traits in cereal crops. LCP lab offers flexibility with the choice of the imaging equipment and the analysis pipeline. It could be a suitable alternative for performing small-scale phenotyping projects or for smaller laboratories or academic institutions with limited resources. Having access to a high-end high-throughput phenotyping facility is still a bottleneck, and thus, a low-cost portable system can help circumvent such limitations.

## Figures and Tables

**Table 1:** Summary of results from the three software programs. Coefficient of determination (*R*^2^) obtained from simple linear regression from projected leaf area (PA) from each software programs and the dry weight of the plants. *R*^2^ of relative growth rate is obtained by regression of dry weight RGR (RGR_DW_) and the projected area RGR (RGR_PA_).

	10 days	20 days	*R* ^2^ of relative growth rate
HTPheno	0.90	0.94	0.90
PlantCV	0.88	0.91	0.89
EasyLeafArea	0.81	0.91	0.86

**Table 2:** Results from HTPheno software for images from different combinations of angles and coefficient of determination (*R*^2^) obtained from simple linear regression between the projected leaf area (PA) and the dry weight of the plants.

	10 days	20 days
Side 1	0.76	0.85
Side 2	0.80	0.83
Top	0.42	0.72
Side 1 + side 2	0.88	0.89
Side 1 + top	0.77	0.91
Side 1 + side 2 + top	0.90	0.94

## Supplementary Materials

Supplementary Materials Description of the two files: Supplementary File 1: R script for preparing barcodes. Supplementary File 2: script for the software digiCamControl for imaging.
