# Supplementary material for: Affordable Imaging Lab for Noninvasive Analysis of Biomass and Early Vigour in Cereal Crops
Source: Biomed Res Int. 2018 Apr 19;2018:5713158. doi: 10.1155/2018/5713158 (PMC5933035; doi:10.1155/2018/5713158)
Supplement: Supplementary Materials — Description of the two files: Supplementary File 1: R script for preparing barcodes. Supplementary File 2: script for the software digiCamControl for imaging. [file 5713158.f1.pdf]

Supplementary File 1: R script for preparing barcodes.

```
#Sticker printing size
#Size& Margin: No margins, fit into given physical size 68x30 millimeter for resolution 300dpi

library(grid)
library(png)

path_to_barcodes <- getwd()
output_filename <- paste0(Sys.Date(),"_", "barcodes.pdf")
barcode_files <- list.files(path_to_barcodes, pattern = "*.png|*.PNG", full.names = TRUE)

#def.par <- par(no.readonly = TRUE) # save default, for resetting...
# Initiate PDF device:

pdf(file = output_filename, onefile = TRUE, paper = 'a4', width = 8.27, height = 11.69, pagecentre = F)
par(mar=c(1,0.2,0.2,0),omi=c(0.1,0,1.1,0),xpd=NA)
par( mfrow = c(7,3) )
for(barcode in barcode_files){
  # Read png barcode:
  png <- png::readPNG(barcode)
  # Create dummy plot:
  plot(1:2,type="n",xlim=c(0,2),ylim=c(0,1),xaxt="n",yaxt="n",xlab="",ylab="", bty="n")
  # Plot png on dummy plot:
  rasterImage(png,0,0,2.2,1)
}

dev.off()
```

Supplementary File 2: Script for the software Digicamcontrol for imaging.

```
<!—The file must be saved with the extension “.dccscript”. -->
<?xml version="1.0" encoding="UTF-8"?>
<dccscript>
  <commands>
    <loop loopcount="1">
                <selectcamera cameranum="0"/>
                <wait time="0" for="camera"/>
                                <capture/>
                                <wait time="1" for="camera"/>
                <selectcamera cameranum="1"/>
                                <capture/>
                                <wait time="3" for="camera"/>¨
                <selectcamera cameranum="0"/>
                                <capture/>
                                <wait time="3" for="camera"/>¨
                <selectcamera cameranum="0"/>
                                <capture/>
                                <wait time="3" for="camera"/>¨
                <selectcamera cameranum="0"/>
                                <capture/>


                </loop>
  </commands>
</dccscript>
```
